# Generalist solutions to complex problems: generating practice-based evidence - the example of managing multi-morbidity

**DOI:** 10.1186/1471-2296-14-112

**Published:** 2013-08-07

**Authors:** Joanne Reeve, Tom Blakeman, George K Freeman, Larry A Green, Paul A James, Peter Lucassen, Carmel M Martin, Joachim P Sturmberg, Chris van Weel

**Affiliations:** 1 University of Liverpool, B122 Waterhouse Buildings, 1-5 Brownlow St, Liverpool L693GL, UK; 2 University of Manchester, Williamson Building, Oxford Road, Manchester M13 9PL, UK; 3 Imperial College London, 25 St James Close, Pangbourne Reading RG8 7AP, UK; 4 University of Colorado, Denver, USA; 5 University of Iowa, 200 Hawkins Drive, Des Moines, Iowa, USA; 6 Nijmegen Medical Centre, Radboud University, Po Box 9010, Nijmegen, The Netherlands; 7 Trinity College Dublin, Rathbourne Road, Dublin 15, Ireland; 8 The Newcastle University, University Drive, Callaghan, NSW 2308, Australia; 9 Radboud University Nijmegen Medical Centre, The Netherlands; Australian Primary Health Care Research Institute, Australian National University, Po Box 9010, Nijmegen Canberra, Australia

**Keywords:** Generalism, Primary care, Action research, Multimorbidity

## Abstract

**Background:**

A growing proportion of people are living with long term conditions. The majority have more than one. Dealing with multi-morbidity is a complex problem for health systems: for those designing and implementing healthcare as well as for those providing the evidence informing practice. Yet the concept of multi-morbidity (the presence of >2 diseases) is a product of the design of health care systems which define health care need on the basis of disease status. So does the solution lie in an alternative model of healthcare?

**Discussion:**

Strengthening generalist practice has been proposed as part of the solution to tackling multi-morbidity. Generalism is a professional philosophy of practice, deeply known to many practitioners, and described as expertise in whole person medicine. But generalism lacks the evidence base needed by policy makers and planners to support service redesign. The challenge is to fill this practice-research gap in order to critically explore if and when generalist care offers a robust alternative to management of this complex problem.

We need practice-based evidence to fill this gap. By recognising generalist practice as a ‘complex intervention’ (intervening in a complex system), we outline an approach to evaluate impact using action-research principles. We highlight the implications for those who both commission and undertake research in order to tackle this problem.

**Summary:**

Answers to the complex problem of multi-morbidity won’t come from doing more of the same. We need to change systems of care, and so the systems for generating evidence to support that care. This paper contributes to that work through outlining a process for generating practice-based evidence of generalist solutions to the complex problem of person-centred care for people with multi-morbidity.

## Background

Strengthening generalist practice has been mooted as part of the solution to emerging problems facing health systems [1-3]. Generalism refers to a professional philosophy of practice deeply known to practising physicians but which is less well understood (and valued) by other stakeholders in the wider health care community. Two approaches to strengthening generalism can be envisaged. Firstly, a professional development approach would see leadership from professional bodies in advocating for change to support and enhance generalist practice. A number of international Colleges have started this work [2,4]. We may complement and extend that work using a research-based approach: to develop evidence of if and when whole-person generalist care offers something different and better to other models of care (for example, condition-focused specialist care). The aim of this paper is to develop a framework to support this second route. We use a key challenge for health systems – managing multi-morbidity – as a critical case within which to describe our ideas. And finish by considering how the work might be used more widely to strengthen generalist practice and so contribute to improving the effectiveness of health care.

“The current care systems cannot do the job. Trying harder will not work, changing systems of care will” [5].

The need to ‘Think Differently’ about care for people with multi-morbidity is an identified priority for health systems across the world [6-9]. Around, one in four people live with a long-term condition; the majority with more than one [9,10]. 70% of health service spending in the UK, for example, is on dealing with long term conditions [6,7]. Multi-morbidity is more common amongst deprived populations [10]. We face an ‘epidemic of multi-morbidity and rising complexity of health needs’ [6,11] resulting from changing demographics and global circumstances. Tackling this problem is a major and urgent strategic priority.

There is a growing mismatch between the needs of populations of people living with complex, chronic illness [10]; and the resources offered by a health system that is focused on condition defined care^a^[12]. Current strategies for managing long term conditions emphasise two elements: delivering interventions defined by evidence-based condition-specific protocols of best practice; and developing teams of multiple professionals to manage the level and complexity of demand thus created [13]. The goal is integrated, effective care [14]. Yet critical review of the current evidence of the impact of integrated care highlights demonstrable improvements in clinical governance (e.g. adherence to protocols), but little impact on patient outcomes or costs to health services [15]. The focus on organisational integration has produced limited benefit for individual patients [15]. However policy makers lack evidence defining high quality alternatives that achieve better patient outcomes.

The Word Health Organisation has called for work to refocus health care to recognise and address the health goals of individuals [16]; changing clinical practice from condition focused care to “individually tailored decision making” [17]. The challenge for health systems is how to develop a model of integrated care that is centred on the needs of the person, rather than the needs of the health system itself [6,18,19]). Medical generalism, or ‘expertise in whole person medicine’, has been proposed as a professional practice-based solution to the problem [2]. However generalism is a professional philosophy of practice [20], rather than the evidence-based model of care needed by policy makers and planners to support service re-design.

Roland & Paddison argued that if health systems are to tackle the challenge of multi-morbidity, clinicians need to be free to exercise professional judgement in their management of patients [21] – to go ‘beyond the protocol’. Recognising that professionals bring their own ‘evidence’ to the decision making process [22], which they integrate with evidence from scientific study to make an informed judgement. This role is core to the expertise of the generalist [1,2,20,23].

**Figure 1:**
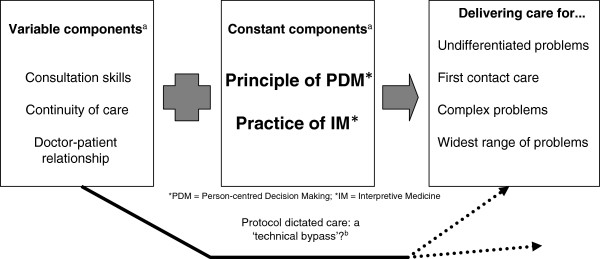
**Defining expert generalist practice [**[26]**,**[27]**].**

Whilst authors have recognised the limitations of protocol-defined care [21,24], as yet there is a lack of evidence to demonstrate that the professional judgement of generalist expertise delivers better outcomes (see Figure 1). Developing generalist solutions to complex problems such as multi-morbidity is an example of a ‘practice-to-evidence gap’ [25]. To date, work has focused on how to get evidence into practice – to address a perceived evidence-to-practice gap and encourage practitioners to use evidence. Our discussions recognise an alternative problem – a gap between the practice and the science, with a need to develop practice-based evidence [25] of the impact of generalist care.

## Discussion

To tackle this practice-evidence gap, we consider how to develop evidence of the impact of generalist practice. We view ‘doing generalism’ as an intervention and so use the Medical Research Council Complex Interventions framework as a guide to evaluating the approach [28]. We must therefore start with a definition.

### Defining the intervention: the practice of generalist expertise

To date, definitions of generalist expertise takes the form of professional expert accounts [1,29] or consensus statements derived from review of professional writing [2,29]. They describe the breadth of the work of the generalist. But lack a focus on the *distinct* expertise of the generalist: that which is unique to and distinguishes generalist care from other approaches to practice.

The recent UK Royal College of General Practitioners report, for example, describes five elements of generalist practice in the primary care context: whole-istic care (seeing the person as a whole and in context; using this perspective as part of one’s therapeutic approach); being able to deal with the widest range of patients and conditions, including undifferentiated problems; taking continuity of responsibility; and coordinating care across health and social care organisations [2]. The importance of the consultation and consultation skills are a recognised component in this and other accounts.

However a specialist (offering condition focused care) may take continuity of responsibility, coordinate care across organisations, and have excellent consultation skills. Generalist expertise can be employed in the first consultation with a patient. These factors may be seen as ‘enablers’ of generalist care, but they do not define generalist expertise. It is the whole-person focus that is defining. But we need to go further in defining the distinct expertise that translates this whole-istic ‘view’ into a ‘therapeutic approach’.

Based on analysis of patients’ accounts of health care experience, Reeve et al. argue that it is not enough to offer personal care (care which is empathic, ongoing and coordinates care beyond disease management) but must go further to offering personalised care [30]. In their study, patients described receiving excellent *personal care* from helpful, concerned and compassionate professionals; who recognised needs beyond disease management, coordinating support for the psychological and practical impact of their illness. However patients also described that their care was not *personalised*. Professionals did not use this whole-person view to influence decisions that were made about their medical care. As a result patients described feeling “trapped on a conveyor belt” with health care becoming an additional burden on, rather than a resource for, living [30].

We therefore propose that the distinct and defining component of the generalist, whole-person focused therapeutic approach, is defined by the *principle* of person-centred decision making which recognises health as a resource for living and not an end in itself [23]. Which is in turn underpinned by the *practice* of interpretive medicine: integrating multiple sources of knowledge (including biomedical, biographical and professional) in a dynamic exploration and interpretation of individual illness experience [31]. Practice leads to decisions about what is wrong, and what is needed to intervene, which support an outcome of health as a resource for living [23,31].

We summarise this understanding of expert generalist practice in Figure 1.

### Evaluating the intervention

With a definition of the intervention that is generalist expertise, it is now possible to consider how we might evaluate the impact of care.

Developing the expertise of generalist practice is part of the core training of Family Physicians and General Practitioners. It is already being delivered to some patients with multi-morbdity, some of the time [32]. We therefore propose to use action research principles to evaluate the impact of care: to identify opportunities to enhance the practice of generalist expertise; to assess the impact of change; and so to generate our practice-based evidence. We start by considering how we would assess capacity for delivering care underpinned by the Expertise of Generalist Practice (EGP).

### What is needed to deliver care underpinned by generalist expertise

Expert generalist practice needs practitioners trained in both the principles (values) and practice (skills) of interpretive practice. Expert generalist practice takes place in the interaction between a patient and a practitioner. However, trained practitioners are not enough to deliver generalist care. External factors also influence practice (Figure 1). We need a whole system approach to recognising, and subsequently evaluating, the care that results from intervening in these complex processes. To help us with this, we draw on the work of May and colleagues and their account of Normalisation Process Theory [33].

Normalisation Process Theory (NPT) draws on extensive research into understanding how interventions into complex systems become part of everyday care. It offers a structure for understanding the processes underpinning care, which enable or constrain the embedding and integration of a set of practices into routine care [34]. Here, we use NPT to understand factors which may support or undermine the continuation of the practice of generalist expertise within the primary care context. When applied to the intervention that is delivery of the expertise of generalist practice, NPT tells us that continued integration needs sustained effort by all parties to ensure that EGP is understood by all (patients, practitioners and the wider health system) and is reinforced through ongoing feedback. Successful integration also needs all parties to both engage with EGP, and to deliver it. This can be summarised as sustained effort in four domains of work: sense making, engagement, action and monitoring [33].

### Outlining Normalisation Process Theory (NPT)

NPT [33] proposes that a complex intervention only becomes a part of everyday practice as a result of significant work and continuous investment by all parties involved.

Integration of a complex intervention into everyday practice depends on successful action within each of four domains of work: namely, sense making, engagement, action and monitoring. We can understand capacity for expert generalist encounters through a consideration of whole system enablers and constraints within each of these four domains.

SENSE-MAKING WORK (also referred to as *Coherence*): refers to the work that people do individually and collectively to understand and make sense of the complex intervention (CI). The CI must be distinguishable from other approaches to care. Individuals and the collective group need a (shared) understanding of the aims, objectives and intended benefits of the CI. The value and benefits of the CI must be internalised – people need a sense of why it matters.

ENGAGEMENT (also referred to as *Cognitive Participation*): looks at the relationship work that people do to start up and enrol in a CI. And also considers how people sustain engagement through legitimating the work and collectively defining the actions needed to sustain practice.

ACTION (also referred to as *Collective Action*): looks at the ‘operational work’ people do to make a set of practices happen. This looks at issues such as managing skill sets, resource allocation, building accountability and trust, and the interactional work with other actors in the health care setting.

MONITORING (or *Reflexive Monitoring*): refers to the appraisal work that people do to understand and assess the CI. It includes the practices used to collect data on effectiveness, the individual and collective work to interpret the data and thus appraise impact and effectiveness; along with the work to modify the CI in light of reflections.

CONTINUOUS INVESTMENT is a fifth dimension which cuts across all 4 domains.

We can use this model to help us develop a whole-system understanding of what is needed to support the practice of generalist expertise in the care of people with multi-morbidity: assessing patient, practitioner and system-level factors which enhance or limit capacity.

#### Assessing capacity for the practice of generalist expertise in the management of multi-morbidity

The table offers a whole-system consideration of what would be needed to support EGP for people with multi-morbidity (Table 1).

**Table 1:** Assessing capacity for expert generalist practice in the management of multi-morbidity

	**Patient factors**	**Practitioner factors**	**Health system factors**	Is investment sustained*?
Sense making	Understand illness as a personal challenge [30] in which they are an active partner (rather than passive recipient of technical fix)	Value personalised decision making and the effort of interpretive practice	Policy and strategy recognise	Training of next generation
				Organisational memory
Engagement	Able and willing to access expert generalist care	Able and willing to make space within working practice to engage in EGP	Policy and organisational systems designed to recognise multi-morbidity as needing personalised care	Continuity of service and care
Action	Patients with the energy and resource to be active partners	Practitioners have skills and resources for interpretive practice (access to range of knowledge including through communication skills, time and support for interpretation and critical review)	System design creates time, space and resource for actions	Stability of service
Monitoring	Patient feedback recognises impact of care on health as a resource for living	Personal and collective professional reflection supports the critical analysis of judgements made in personalised decision making	Quality markers and performance management recognise EGP	Feedback integrated into ongoing service development – action learning principles

* robust enough to last within a changing healthcare context.

It is clear that we will need to draw on multiple sources of data including policy/strategy review, audit of care, and stakeholder interviews with patients and practitioners if we are to assess capacity for EGP in the management of multi-morbidity. We have published [32] and (as yet) unpublished data from the UK which suggests enablers for expert generalist care include: patient and professional desire for personalised care for people with multi-morbidity (sense making), supported by strong interpersonal relationships between patients and their health professionals (action). But constraints include a shortage of skills and confidence in interpretive practice along with a lack of time for a comprehensive personal assessment of need (engagement and action). Also a lack of feedback on why it matters (monitoring) supporting the ongoing effort needed to deliver personalised expert generalist care.

### What is the impact of expert generalist care: evaluating generalism in action

A structured assessment of capacity for expert generalist practice allows us to identify what is needed to strengthen the consistent delivery of quality generalist care to people with multi-morbidity. By intervening to address those needs and evaluating the impact of the changes, we start to generate practice-based evidence of EGP. This is the process of action research.

### Action research

Action research is a process whereby people work together, and learn together, whilst tackling a real problem. People acquire new knowledge through actual practice, underpinned by critical reflection on the process and outcomes of practice [35]. Action learning makes explicit the tacit knowledge of practice [35]. It is a dynamic model by which practice evolves based on critical reflection and continual learning. It thus offers an approach whereby we can develop evidence out of everyday practice – practice-based evidence [25].

**Figure 2:**
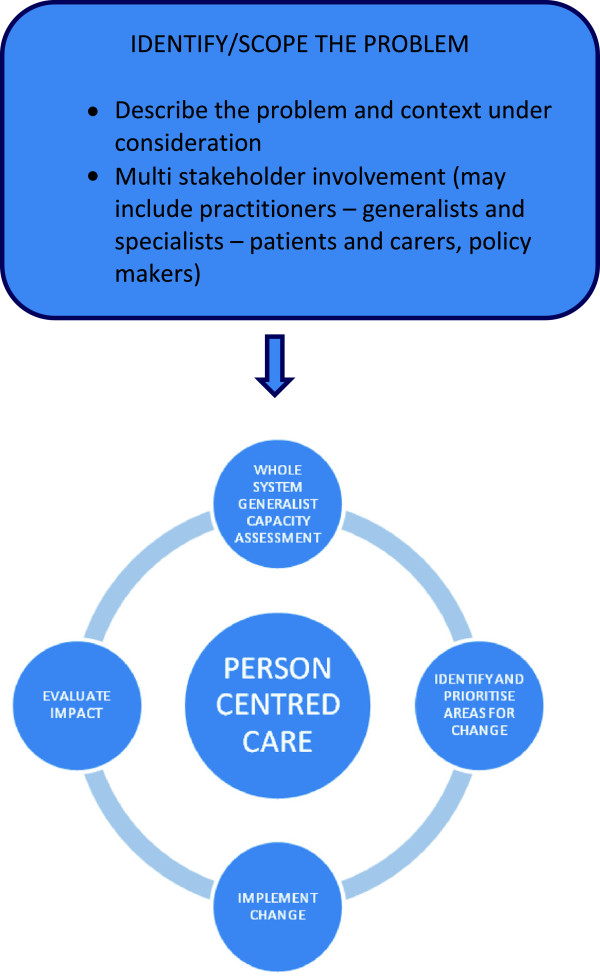
The generalism in action framework.

Drawing on these principles of action research, we present the Generalism in Action (GIA) framework as a change model to describe, deliver and evaluate expert generalist practice. The framework focuses on developing and evaluating generalist solutions to health care problems within a given context. It supports the user in asking ‘(how) can an expert generalist approach enhance person-centred primary care within the context of particular area or problem?’ It can therefore be used to critically enhance and evaluate EGP for the care of people with multi-morbidity (Figure 2).

To use the GIA framework, we start with a description of the problem. In this case, we are considering the care of people with multi-morbidity. Next we assess capacity for expert generalist encounters, systematically identifying areas of potential need for change. We can then pilot changes: implementing *and evaluating* the impact of change using action learning principles. We make the tacit knowledge [22] used by generalist practitioners to guide person-centred decision making transparent, and thus open to critical review. We generate evidence of if and when expert generalist care makes a difference. We therefore build an evidence-base of practice, practice-based evidence [25], supporting future practice and policy development through describing if and when generalist care ‘matters’.

#### An example of developing action research for multi-morbidity

Reeve & Bancroft describe an early example of the application of the GIA idea in the form of a service development project in a UK practice [32,36]. The Practice team identified a problem: a spiralling burden of workload for patients and practitioners in dealing with the needs of housebound patients with multi-morbidity. The team felt that patients were not accessing expert generalist care, largely because systems of care were organised around the technical delivery of specialist-defined protocols of care. Changes were made to offer protected time for expert generalist care of a sample of patients identified as being at greatest risk. Evaluation of impact revealed that the new care model resulted in changes to care planning for around two thirds of patients. However care for the remaining third was no different to that delivered by standard protocol-based chronic disease management. Findings from this initial practice based study have informed stakeholder discussions leading to development of a formal research proposal to critically develop and evaluate the learning – to develop practice-based evidence of the impact of changes to care.

### The Complex Needs Project at Vauxhall Primary Health Centre

**Delivering EGP.** The Complex Needs project at Vauxhall Primary Health Centre (VPHC).

VPHC is a medium sized General Practice in a deprived area of Liverpool. The team were concerned that housebound patients with complex illness (multimorbidity) had limited exposure to expert generalist care. Patients received high quality chronic disease management care delivered at home visits by nursing staff with excellent consultation skills (as assessed by the performance framework that is QOF and patient surveys). However, GP care was predominantly reactive in the face of acute illness.

The clinical team asked, what would be the impact of changing from usual protocol-defined chronic disease management care to expert generalist care for housebound patients with multimorbidity?

They established a register of housebound patients with more than 2 long term conditions and on 5 or more medicines a day. Changes to care involved introducing dedicated time for a GP home visit. GPs with expertise in generalist practice undertook an assessment of the impact of health, illness, and healthcare on individual daily living. Visits took about an hour; and were repeated as many times as needed for health needs to stabilise (where continued visiting made no further changes to care). The most common changes to care were to reduce the burden of health care: reduced prescribing, surveillance, exempting the patient from protocol defined care pathways.

Review of care articulated a number of core components in the delivery of generalist expertise:

Justification of medicalisation of health and illness experience for this individual at this time. (A *shift from a specialist view of health need asking, does this patient meet the diagnostic criteria for a given disease, and if so is it appropriate (safe) to treat? The onus is on justifying exempting an individual from disease-defined care. To an expert generalist approach: asking (how) does a disease-model help us understand and address the health and illness needs of this individual and at this time? The onus is on justifying a decision to instigate medical intervention for an illness problem*[32])

A principle of Minimally Disruptive Medicine [37]: minimising the burden of health care, as well as illness, on daily living

Continuity of episode to support interpretive practice: having the same named practitioner responsible for the interpretive care of an individual for at least the current episode of care (until care needs had stabilised). Including the use of good clinical notekeeping/summaries to support continuity.

The team recognised the need for further work in other settings to refine these identified core components

## Summary

In western communities, health needs increasingly reflect the rise in prevalence of chronic complex illness. We need to adapt health systems to meet this need; through providing a balance of generalist and specialist care [1,16]. We have an increasingly detailed understanding of the pathology of the common chronic diseases facing our communities, and so of ways to intervene in the pathological process. Guidelines for care derived from ‘robust synthesis of clinical and economic evidence’ support optimisation of disease-focused care [24]. However, we lack an equivalent framework supporting generalist care. We lack practice-based evidence to demonstrate the utility and trustworthiness of generalist care alongside the better described specialist approach. We have discussed an example of improving care for people with multi-morbidity, but suggest that the approach may be of utility for a range of ‘complex problems’ and areas of diagnostic uncertainty for example, medically unexplained symptoms.

We have outlined an approach to address this practice-evidence gap based on action research principles*.* Action research for quality improvement is not a new concept in health care [35]; but evidence for practice is still traditionally dominated by experimental designs. Yet a number of sources are challenging a ‘drainpipe’ model of knowledge translation, which sees research evidence as a way to inform practitioners and policy makers what they ‘should’ be doing [25,38,39]. There has been a call for more critical examination of practice, based on the principles of participatory research and action learning, in order to support the translation of practice into an evidence base which contributes to the critical development, delivery and evaluation of quality care [25]. This is particularly appropriate when the expertise lies within the clinical community.

If this approach is to be successful, we need also to recognise and address the barriers to undertaking this sort of research - including issues related to funding, career progression for action researchers and support for dissemination. Critical implementation and evaluation of the GIA framework may also provide us with evidence of the value and limitations of action research for improving health care.

We thus describe a new framework to support the generation of practice-based evidence of the impact of individually tailored generalist care. We have evidence supporting the development of the component parts, but now seek to evaluate whether the framework can support change towards more person-centred care. Testing the framework is one goal for a new Special Interest Group (SIG) within NAPRCG (the North American Primary Care Research Group). The Generalism SIG was formed in 2011 to support the critical development, delivery and evaluation of generalist care within a modern managed health care context. It is an international collaboration with representatives from North America, Australia and Europe. Developing and implementing the GIA is one area of our work. We welcome comments, feedback and expressions of interest in joining us in this work.

## Endnote

^a^ We refer to ‘condition’ rather than ‘disease’ defined care since: health policy refers predominantly to long term conditions, rather than diseases; dealing with the management of a ‘condition’ that ‘warrants attention’ – for example hypertension – rather than a disease per se.

## Abbreviations

EGP: Expert Generalist Practice – the practice of generalist expertise; NPT: Normalisation Process Theory; GIA: Generalism in Action; NAPCRG: North American Primary Care Research Group.

## Competing interests

The authors declare that they have no competing interests.

## Authors’ contributions

This paper arises from discussions at the inaugural NAPCRG Generalism Special Interest Group meeting held in Banff in 2011. JR conceived the paper and led the writing of the manuscript. TB, GF, LG, PJ, JM, CM, JS & CvW contributed substantially to the intellectual content of the paper through providing critical commentary, interpretation and revisions. All authors read and approved the final manuscript.

## Authors’ information

All are members of the NAPRCG Generalism Special Interest Group. JR holds a National Institute of Health Research Clinician Scientist Award supporting a body of work on Generalist Solutions for Complex Problems. GF was a member of the Royal College of General Practitioners (UK) Commission on Generalism.

## Pre-publication history

The pre-publication history for this paper can be accessed here:

http://www.biomedcentral.com/1471-2296/14/112/prepub
